# Comprehensive Survey of Plagiarism in Iran

**DOI:** 10.12669/pjms.36.7.3456

**Published:** 2020

**Authors:** Mohammad Bagher Rokni, Negar Bizhani, Farrokh Habibzadeh, Dariush Daneshvar Farhud, Neda Mohammadi, Ahad Alizadeh, Ladan Rokni

**Affiliations:** 1 Mohammad Bagher Rokni, PhD, Department of Basic Sciences, Iranian Academy of Medical Sciences, Tehran, Iran Department of Medical Parasitology and Mycology, School of Public Health, Tehran University of Medical Sciences, Tehran, Iran; 2 Negar BIZHANI, PhD Candidate, Department of Medical Parasitology and Mycology, School of Public Health, Tehran University of Medical Sciences, Tehran, Iran; 3 Farrokh Habibzadeh, MD, R&D Headquarters, Petroleum Industry Health Organization, Shiraz, Iran; 4 Dariush Daneshvar Farhud, MD, PhD, Department of Basic Sciences, Iranian Academy of Medical Sciences, Tehran, Iran; 5 Neda Mohammadi, Department of Epidemiology and Biostatistics, School of Public Health, Tehran University of Medical Sciences, Tehran, Iran; 6 Ahad Alizadeh, PhD, Department of Epidemiology and Biostatistics, School of Public Health, Tehran University of Medical Sciences, Tehran, Iran; 7 Ladan Rokni, PhD, Asia Contents Institute, Konkuk University, Seoul, South Korea

**Keywords:** Plagiarism, Scientific Misconduct, Publication ethics, Iran

## Abstract

**Background and Objective:**

We conducted this study to assess the prevalence of plagiarism and to shed light on some dark aspects of this issue. The main objectives included to find out the etiology, prevalence, and detection of various forms plagiarism.

**Methods:**

In this Cross-sectional study we used a questionnaire, face-to-face interview, analyzing the present notifications and codes, websites, and literature review. The current study was conducted throughout Iran from 2017-2018. Those associated with scientific journalism, academic staffs, and authors were interviewed or asked to fill out a prepared questionnaire.

**Results:**

Nine hundred seventy nine questionnaires were circulated. Out of this 706 (72.1%) were completed and returned. Those with a master degree were most cooperative in filling out the questionnaires (36.4%); followed by Assistant Professors (29.6%). About 74.1% of respondents, had not participated in any educational workshops on plagiarism (*P*<0.001) while 10.8% had not heard anything about plagiarism (*P*<0.001). As regards correct reply as for definition and detecting plagiarism; 91.1%, 40.8%, 48.4% and 57.9% could reply correctly (*P*<0.001). Forty-one-point one percent of the participants believed that reprimand would be the best punishment. The percentage of plagiarism as per people associated in journal administration, was 22.9%; based on experts’ opinions, it was 30.0%; and based on analysis of some journals published in Iran, it was 25.5%.

**Conclusion:**

We found a noticeable prevalence of plagiarism in Iran. Many factors are involved in this misconduct; most important being the need for academic staff and students to publish e more papers regardless of their quality to meet some of the academic requirements. Considering the high rank of Iran in terms of scientific growth worldwide, it is expected from the regulatory authorities to monitor all aspects of scientific misconducts in medical journalism.

## INTRODUCTION

According to Merriam Webster dictionary, “plagiarism” is defined as “to commit literary theft: present as new and original an idea or product derived from an existing source”.^1^ Iran, like many other countries, has been faced with plagiarism in academic centers. Some international bodies have pointed out plagiarism promoted by authors, researchers in Iran, even by those occupying coveted positions at administration levels.^2^

Some investigations have been conducted in Iran to find out the frequency and causes of plagiarism.^3^-^7^ In a study conducted in Tehran, 11.6% of academic staff members could reply correctly to questions on plagiarism.^3^ only 14% of PhD students in Tehran, could respond correctly to questions regarding the plagiarism, which reflected lack of enough training into this arena.^4^ In Iran, for academic carrier promotion it is essential to publish a number of papers in journals indexed in ISI. For defending PhD dissertations, one paper is essential. These two prerequisites are mentioned as most important causes of committing plagiarism in Iran.^5^ In Hamadan, western Iran, 38% of people confessed that they had committed plagiarism at least once during their academic career.^6^ In another study, publication misconducts, was identified in 4.9% of the Iranian academic authors.^7^

Plagiarism has been reported more or less not only in Iran, but also in many other countries, especially those where English is not their mother tongue. Studies have been reported from Pakistan^8^, USA^9^, UK^10^, Indonesia^11^, Thailand^12^, and Taiwan.^13^ These studies show that plagiarism occurs even in countries where English is a native language. However, the causes are intricate and need an extensive survey to be conducted throughout the country. We therefore conducted the current study to highlight dark aspects of plagiarism in Iran.

## METHODS

This study was conducted after approval by the Iranian Academy of Medical Sciences. (Ref.7765/1 / f / a / c dated 25/8/94). The methodology used for collecting data was by filling out questioners, face-to-face interview, analyzing the present notifications and codes besides analyzing the frequency of plagiarism in journals published in Iran. The study was conducted during 2017 to 2018. People associated with journalism, academic staffs, and authors were included .The study covered all provinces in Iran.

### Circulation of Questionnaire

Based on the statistician recommendation, for the first category, we circulated 979 questionnaires throughout the country amongst the academic staff, students, and personnel working in scientific journal offices. The reliability and validity of the questionnaire had already been approved by Poorolajal et al.^6^ We enrolled only those participants who had already contributed by publishing at least one paper. The questionnaire consisted of four sections including general characterization of participants, appraisal of their knowledge, attitude and practice.

### Face-to-face interview

The next category included a face-to-face interview with distinguished experts in medical journalism. They were selected based on the good history of having academic publication or authorities of high ranking in the country involved with scientific publishing. Altogether nine experts and people associated with regulatory authorities were interviewed.

### Analyzing the present notifications and codes

All regulations and codes found in various universities, Ministry of Health and Medical Education, and the Ministry of Sciences, Research and Technology, were reviewed and analyzed.

### Survey of the domestic journals

The third category was studying papers published in journals where the first author had good cooperation, as an editor. In all, 11 journals all published in English were reviewed which included the following:

1. Iranian Journal of Public Health
2. Iranian Journal of Parasitology
3. International Journal of Occupational Hygiene
4. Iranian Journal of Toxicology
5. Iranian Journal of Child Neurology
6. Iranian Journal of Pathology
7. Journal of Research in Health Sciences
8. Journal of Arthropod-Borne Diseases
9. Health Promotion Perspectives
10. Journal of Iranian Clinical Research
11. Journal of Chemical Health Risks

### Statistics analysis

SPSS^®^ for Windows^®^ version 21 (Chicago, IL, USA) was used to analyze the data. A *P* value <0.05 was considered statistically significant. χ^2^ and Fisher exact tests, when appropriate, were used to assess the association between qualitative variables. All 95% confidence intervals (CI) were estimated using the test of proportion.

## RESULTS

From 979 circulated questionnaires, 706 were completed and returned, giving a response rate of 72.1%. Those with a master degree were the most cooperative group in filling out the questionnaires (36.4%). In terms of academic rank, Assistant Professors had the most cooperation (29.6%) (Data not presentenced). Among those associated with journal work, reviewers filled out questionnaires more than others (32.3%). Those associated with Ministry of Health (84.7%) ranked first in filling out the questionnaires. Half of those who responded had published on an average seven papers in English and four in Persian language.

When asked the participants to answer questions regarding various aspects of plagiarism 74.1% of respondents, had not attended any educational workshops on plagiarism (*P*<0.001) and 10.8% had not heard anything about plagiarism (*P*<0.001). The frequencies of correct answers to four questions on the definition and detecting plagiarism were 91.1%, 40.8%, 48.4% and 57.9%, respectively (Table-I).

**Table-I:** Distribution of participants’ opinion on questions in relation to correct detection of plagiarism.

Question	Correct answere		Wrong answer		P value

	No.	%	No.	%	
Which of the following practices may be considered as plagiarism?	631	91.1	62	8.9	<0.001
A) The author turns another’s idea as his or her own					
B) The author turns another’s text as his or her own					
C) The author turns another’s photo or figure as his or her own					
D) All choices					
Which of the following practices may NOT be regarded as plagiarism?	272	40.8	402	59.2	<0.001
A) The author alters text without credit					
B) The author alters text with credit					
C) The author does not alter text without credit					
D) The author does not alter text with credit					
In what way reprint of one’s own previous work may NOT be considered plagiarism?	310	48.4	330	51.6	0.452
A) In the same language with permission from the previous and new publisher					
B) In the same language without permission from the previous and new publisher					
C) In another language with permission from the previous and new publisher					
D) In another language without permission from the previous and new publisher					
In what way coping another’s work, word-for-word, may NOT be regarded as plagiarism?	390	57.9	284	42.1	<0.001
A) The author copies a few phrases without citing to sources					
B) The author copies a few phrases with quotations and cites to sources properly					
C) There is no limitation if the author cites to sources properly					
D) There is no limitation if the author cites to sources properly and uses quotations					

**Fig.1:**
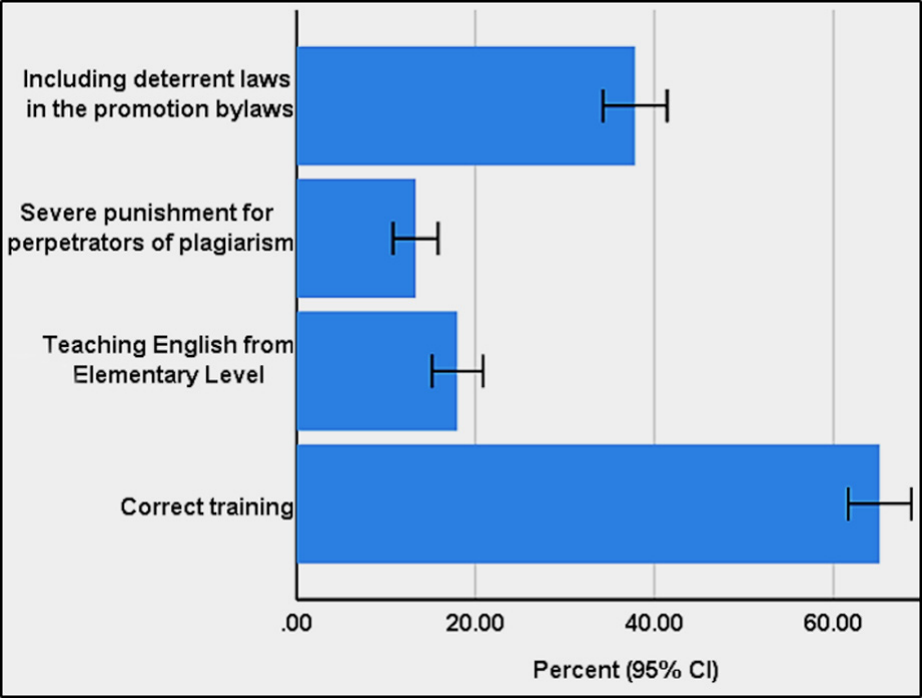
The participants’ views about the best method for prevention of plagiarism. Error bars represent the 95% CI.

As regards the respondents’ view on the level and kind of punishment for committing plagiarism, most of the participants (41.1%, 95% CI: 37.4% to 44.8%) believed that reprimand would be the best punishment. The person should also be asked to attend some educational workshop (38.5%, 95% CI: 34.9% to 42.2%).On scientific misconduct some participants believed that the name of those who committed plagiarism should be included in a black list (26.9%, 95% CI: 23.7% to 30.4%); others believed that they should be prosecuted in a court of law (8.2%, 95% CI: 6.4% to 10.6%). Other punishments mentioned included giving a simple hint (7.2%, 95% CI: 5.5% to 9.5%), removal from academic positon in the university (5.8%, 95% CI: 4.3% to 7.9%), and ignoring the case (0.4%, 95% CI: [0.1% to 1.4%). The participants believed that the best method to prevent plagiarism was “proper education;” the worst was “severe punishment” (Fig.1).

Another important question was about the reasons that compelled the participants to commit plagiarism. 53.4% believed that the most important reason was forcing students and faculty members to publish papers which was followed by national policies to produce more scientific papers as expressed by 44.6% of the participants. People associated with journal administration revealed that they see plagiarism in about 23% in manuscripts submitted to the journal. About 83.6% (95% CI: 80.6% to 86.3%) of those interviewed stated that they had never indulged in plagiarism for their publication. When the participants were asked about their opinion on different aspects of plagiarism including the willingness to commit plagiarism, most of them were reluctant to commit plagiarism (Table-II).

**Table-II:** Distribution of people who filled out the questionnaires based on their personal opinion of rejection or acceptance of plagiarism (all P values <0.001)

Question	Agree (%)	Disagree (%)	No idea (%)
I sometimes get tempted to use the works of others without citation, because others do it too	65 (9.7)	528 (78.9)	76 (11.4)
I think plagiarism is bad because it violates the moral values acceptable to me	620 (92.5)	14 (2.1)	36 (5.4)
I think plagiarism would hurt my scientific credibility	629 (39.2)	19 (2.8)	27 (4)
I think writing a high-quality paper is not possible without plagiarizing another’s work	214 (32.2)	302 (54.4)	149 (22.4)
I think plagiarism a land mark of inability of the author rather than his or her intelligence	518 (77.1)	76 (11.3)	78 (11.6)
I think there is no plagiarism in English-speaking countries	36 (5.3)	509 (75.6)	128 (19)
In one case, deliberate plagiarism led to the dismissal of a professor. My opinion is…	240 (36.9)	240 (36.9)	170 (26.4)

### Analyzing the experts’ opinions

Views of all the experts who were interviewed face-to-face about certain aspects of plagiarism in Iran are shown in Table-III.

**Table-III:** Experts’ opinions on plagiarism in Iran

Question	Answer
Is there a scientific breakthrough in Iran? What is the percentage if yes?	All the experts, without exception, believed that there was undoubtedly a scientific breakthrough in Iran, with an average rate of 30%.
What do you know the cause of this phenomenon in Iran and the tendency of people towards it?	Unawareness of researchers;
	Not knowing how to cite articles;
	People need to publish articles to graduate and people need to publish multiple articles to get carrier promotion;
	Lack of English language proficiency among Iranians
How would you describe the role of English language in leading people to this phenomenon?	Most scholars agreed with the principle of teaching English in schools
What do you know about ways to prevent scientific and literary theft?	Correct education from an early age and proper culture building;
	Holding a writing workshop;
	Also educating teachers at all levels of education;
	Cultivating both through schools and through television and the press; Changing promotion rules;
	Emphasizing on the quality of papers not quantity;
	Strong rules to prevent plagiarism;
	Training into plagiarism in university
What punishment and punishment do you consider appropriate?	Put the person in a black list;
	Do according to the rules of COPE;
	Preventing or delaying one’s career;
	Reducing salary;
	Hint, reprimand and dismissal;
	Forced to attend training courses; etc.

### The frequency of plagiarism in some Iranian journals

The eleven journals were edited by the first author for a long time, hence we had access to the history of plagiarism in these journals. Hence we decided to detect the frequency of plagiarism in their submissions. The expert in the team used Google as the best method to check the plagiarism. iThenticate^®^ was also used.

The average frequency of plagiarism in these journals was 25.5% (range: 10% to 41%). The frequency in journals published in Tehran was lower than those published elsewhere in Iran.

### Analyzing the present notifications and codes

All existing rules concerning academic promotion and plagiarism were reviewed which will be discussed.

## DISCUSSION

We aimed to detect the frequency of plagiarism and determine its various aspects throughout the Iran using a compendium of methods already stated. The results showed that plagiarism is more or less frequent in Iran.

### Frequency of plagiarism in Iran and other countries

The first method of detecting plagiarism was asking the people associated with administration of scientific journals in Iran. They estimated a frequency of 23% on average. Most of them used software which was available free of charge like Small SEO tools (https://smallseotools.com/plagiarism-checker/) or Google to check for plagiarism. Another way was asking the view of experts on journalism which yielded 30% on average. Eventually the last method was checking the frequency of plagiarism in submitted manuscripts to 11 journals published in Iran, which had an average of 25.5% (range: 10% to 41%). In our opinion, taken together, we can expect a frequency of plagiarism between 20% and 25% in Iran. Different studies in Iran have reported the rate of plagiarism as 4.9%^3^, 38%^6^, 31%^14^ and 4.9%.^7^ Of course, all these were detected based on the answers given by participants, which makes it difficult to judge the integrity of the responses. Since plagiarism is notorious, it is possible that some people are reluctant to tell the truth.

Students and researchers are aware of the “unacceptability” of plagiarism. However, they do not know how to prevent it. Continued efforts made by the education system to address this deficiency are useful.^15^ The major reason for the high percentage of plagiarism can be attributed to the lack of a well-designed curriculum and institution of preventive measures against committing plagiarism. There is a significant relationship between students’ knowledge of the academic system where they are learning and doing research and their academic attitudes on plagiarism.^16^

As stated earlier, the frequency of plagiarism in English-speaking countries is not zero because knowing English well is only one of the competencies to write a scientific paper. A study from USA, showed that 46% of the manuscripts submitted to International Journal of Exercise Science contain a form of plagiarism.^17^ Indeed, “30% of submitted manuscripts included plagiarism from a previous publication of the senior author and 16% of submitted manuscripts included plagiarism from another investigator’s work and/or website”.^17^ In Croatia, 34% of students plagiarized less than 10% of the text. The average frequency of plagiarism was 19%.^18^ In Turkey^19^. A study which included 347 students, 94.0% admitted using copy-paste methods to write their papers. About 50.7% did not mention any references and 35.2% had no suitable references. Comparison of original and copied texts showed that 27.1% of the students did not change even the original format while 34.3% rephrased the sentences. Many other studies, more or less, testify that plagiarism is present in nearly all countries of the world to some extent.^13^,^20^-^24^

### The role of education in preventing of plagiarism

An important finding, we noticed in our study was that 74.1% of participants had not been trained to avoid plagiarism. A study conducted in Iran shows that being aware of details of plagiarism and codes of copyright is very important for students to prevent plagiarism.^4^ Nearly all studies have emphasized that knowledge about plagiarism would decrease the frequency of plagiarism.^25^-^27^ One of the recommendations expressed by the panel of experts in our study was that inclusion of an academic course on plagiarism in undergraduate and postgraduate curricula would be beneficial. A researcher in an operational project, transformed the existing management approach based on the “punitive attitude” into an “educational attitude” and considers the practical approach to effective management of plagiarism in the three areas of education, cognition, and policy.^28^

### The motives for committing plagiarism

The best approach to decrease or prevent the frequency of plagiarism, is to identify the motives of plagiarizers. Our study showed that more than half of the participants believed that the most important reason was forcing students and faculty members to publish articles.^29^ An important study conducted in Tehran found that forcing to publish more papers in ISI-indexed journals is the most important reason for committing plagiarism.^5^ Unfortunately, this issue has given birth to many companies which are widely involved in production, sale and purchase of plagiarized papers. 30,31 Another study showed that “the majority of the syllabuses (83.6%) lacked a plagiarism policy and those that did include a policy were often vague in their definition of the phenomenon”.^32^ Fortunately, new rules for academic promotion have been framed which has decreased the role of papers to some extent in promotions which is a welcome step (https://heiatelmi.ir/wp-content/uploads/2017/02/aiinname-erteqa-pezeshki-azad95-heiatelmi.ir_.pdf). In Australia and England, an approach of impact review system for published articles is given weightage instead of the number of published articles.^33^

### Problem of not being fluent in English

Another reason to boost the issue of plagiarism, stated by the experts and participants in our study was not being fluent in English.^34^,^35^ although as stated earlier, plagiarism is also found in countries with English as native language. However, it is obvious that in English for Academic Purpose (EAP) group countries^36^, where English is taught as the 2^nd^ language, this problem is much worse. Most experts in our study stated that teaching in early ages can improve the chance to learn English better. Our experiences on detecting the kind of plagiarism show that the most types of plagiarism in Iran were plagiarism of words.

### How to deal with plagiarizers

Our results showed that 41.1% of participants recommended reprimand as the best punishment for the plagiarizer followed by making him to participate in an educational workshop (38.5%). We do believe that the best approach to deal with a plagiarizer is to implement the codes set by the Committee on Publication Ethics (COPE) (https://publicationethics.org/). This is the best platform to verify the rate of plagiarism as minor and major, and how to deal with it. Unfortunately, there are some contradictions in Iran in terms of dealing with plagiarizers. We have noticed a category of dismissal of a professor to neglecting cases of major plagiarism. We hope announcement of the new law of plagiarism set by the government in Iran can improve the situation and harmonize the efforts made to prevent this phenomenon or at least to decrease its frequency.

### Strengths of the study

For the first time we have conducted a comprehensive study with different approaches to detect various aspects of plagiarism. Detecting the frequency of plagiarism using three approaches assures us of the integrity of the study to a great extent.

### Limitations of the study

It included poor collaboration of participants. We circulated two thousand questionnaires and only about one-third of them responded. The reason for not responding is unknown. To attract more participation the participants were assured of confidentiality and they were asked not to write their names.

##  RECOMMENDATIONS

1. The regulatory authorities looking at the scientific misconduct need to purchase internationally accredited software programs to check plagiarism, and make it available to reputable scientific journals free of charge. At present some of the journals do get this software free but it should be provided to all the journals.
2. A specialized office in universities can check manuscripts for plagiarism before they are being submitted to journals. Some foreign universities do practice this. The existing rules should be amended to reduce the emphasis on the quantity of papers necessary for academic promotion. As long as the academic promotion is linked with the number of papers published, the sale and purchase of papers cannot be checked or eliminated altogether.^37^ It should be replaced with the impact of papers and real citations not the numbers.^38^
3. Students should be educated during their undergraduate studies about scientific misconduct and not during their postgraduate courses. We have noticed in workshops that many students were surprised after they learned the details of plagiarism and its consequences. Many of them as well as faculty members were surprised to know the real situation related to plagiarism. They consider it enough to mention the reference and have no idea about the use of quotation marks.
4. Education about scientific misconduct should be mandatory for Master’s or PhD students’ workshops.
5. Teaching English from elementary school is the best time to learn English.

## CONCLUSION

As expected, prevalence of plagiarism was quite common in Iran. There are various reasons and the most important was the requirement for faculty and students to publish more papers regardless of their quality. As international and national statistics show, the situation of scientific growth of Iran is very good which is commendable as regards the quantity of articles. It is widely believed that Iranian scientists are making significant contribution to the medical literature but it will be unwise to jeopardize this by indulging in committing plagiarism by some authors intentionally or due to ignorance. Educating the authors through their active participation in courses, workshops should be enhanced. They should be motivated to participate in these training workshops.

### Author’s Contribution:

**MBR:** Is corresponding author and takes all responsibility in terms of observing all regularities and rules in relation to integrity and accuracy of the study.

**NB**, designed, collecting data, conducting all face-to-face interviews with experts, and final approval of the galley proof.

**FH**, conceived, designed, manuscript writing, editing and interpretation of the findings, and final approval of the galley proof.

**DDF**, conceived, designed, interpretation of findings.

**NM and AA**, did statistical analysis, writing and editing manuscript, and final approval of the galley proof.

**LR**, collecting data, manuscript writing and editing, and final approval of the galley proof.

All authors agreed to accountable for all aspects of the study in terms of integrity and accuracy.
